# PUMA: PANDA Using MicroRNA Associations

**DOI:** 10.1093/bioinformatics/btaa571

**Published:** 2020-06-17

**Authors:** Marieke L Kuijjer, Maud Fagny, Alessandro Marin, John Quackenbush, Kimberly Glass

**Affiliations:** Centre for Molecular Medicine Norway, University of Oslo, Oslo 0318, Norway; UMR7206 Eco-Anthropologie, Muséum National d’Histoire Naturelle, Centre National de la Recherche Scientifique, Université de Paris, Paris 75016, France; Centre for Computing in Science Education, Department of Physics, University of Oslo, Oslo 0316, Norway; Department of Biostatistics, Harvard T.H. Chan School of Public Health, Boston, MA 02115, USA; Department of Data Science, Dana-Farber Cancer Institute, Boston, MA 02215, USA; Channing Division of Network Medicine, Harvard Medical School, Boston, MA 02115, USA; Channing Division of Network Medicine, Harvard Medical School, Boston, MA 02115, USA

## Abstract

**Motivation:**

Conventional methods to analyze genomic data do not make use of the interplay between multiple factors, such as between microRNAs (miRNAs) and the messenger RNA (mRNA) transcripts they regulate, and thereby often fail to identify the cellular processes that are unique to specific tissues. We developed PUMA (PANDA Using MicroRNA Associations), a computational tool that uses message passing to integrate a prior network of miRNA target predictions with target gene co-expression information to model genome-wide gene regulation by miRNAs. We applied PUMA to 38 tissues from the Genotype-Tissue Expression project, integrating RNA-Seq data with two different miRNA target predictions priors, built on predictions from TargetScan and miRanda, respectively. We found that while target predictions obtained from these two different resources are considerably different, PUMA captures similar tissue-specific miRNA–target regulatory interactions in the different network models. Furthermore, the tissue-specific functions of miRNAs we identified based on regulatory profiles (available at: https://kuijjer.shinyapps.io/puma_gtex/) are highly similar between networks modeled on the two target prediction resources. This indicates that PUMA consistently captures important tissue-specific miRNA regulatory processes. In addition, using PUMA we identified miRNAs regulating important tissue-specific processes that, when mutated, may result in disease development in the same tissue.

**Availability and implementation:**

PUMA is available in C++, MATLAB and Python on GitHub (https://github.com/kuijjerlab and https://netzoo.github.io/).

**Supplementary information:**

Supplementary data are available at *Bioinformatics* online.

## 1 Introduction

The regulation of gene expression involves a complicated network of interacting elements. The biological process of transcription begins with the binding of transcription factors to specific sequence motifs upstream of a gene’s transcription initiation site. This induces conformational changes in the DNA and initiates the assembly of the RNA polymerase complex, which in turn carries out transcription of the gene to a messenger RNA (mRNA). At a post-transcriptional level, small non-coding RNA molecules such as microRNAs (miRNAs) can repress mRNA translation and cause degradation of the mRNA transcript (Williams, 2008). What emerges is not a single set of interactions, or even a single pathway, but a complex network of interacting genes and gene products. Capturing these interactions is critical as we seek to understand how gene expression is regulated in different tissue environments, and how this regulation is disrupted in disease.

miRNAs are small non-coding RNAs of ∼22 bp in length that can bind to the 3′ untranslated region (UTR) of their mRNA targets. The miRNA–mRNA duplex then associates with Argonaute family proteins, which recruit factors that induce mRNA degradation and translational repression (Guo *et al.*, 2010; Ha and Kim, 2014). Most human protein-coding genes are thought to be regulated by miRNAs, with over 60% having conserved miRNA binding sites in their 3′ UTR (Friedman *et al.*, 2009). miRNAs are generally thought to moderately downregulate their target genes, as individual sites usually reduce protein output by <50% (Bartel, 2009). However, most mRNAs have multiple miRNA regulatory sites in their UTR and miRNAs bound to these sites can act additively (Bartel, 2018). In addition, miRNAs may bind to many more non-canonical regulatory sites and act together, thereby increasing their regulatory potential (Kim *et al.*, 2016).

Given the large number of miRNAs in the human genome (currently thought to be ∼2300; Alles et al., 2019) and because of their broad regulatory potential, their regulatory profiles are often modeled in gene regulatory networks (Chen *et al.*, 2019). Such networks have generally been estimated using the inverse correlation between miRNA and mRNA expression levels. However, this approach has its limitations, as many different mechanisms modulate miRNA activity (Gebert and MacRae, 2019) and RNA transcripts may compete for binding to miRNAs, creating a more complex regulatory network than can be captured with co-expression patterns alone (Bosia *et al.*, 2017).

Other methods start with a prior network based on target predictions and then ‘color’, or assign weights to, the network’s nodes based on miRNA and mRNA expression levels (Dragomir *et al.*, 2018). However, target predictions are often different from actual interactions, with many studies reporting positive correlation between miRNAs and about half of their predicted targets (Wang and Li, 2009). Furthermore, target prediction remains challenging, and different algorithms may result in rather different networks of putative interactions (Riffo-Campos *et al.*, 2016). Moreover, some genes may be regulated by a miRNA even though they are not predicted as a target of that miRNA by current prediction algorithms. Such ‘new’ edges cannot be learned if a model only considers known predicted targets.

Here, we present PUMA, or PANDA Using MicroRNA Associations, an algorithm that can directly model robust regulatory edges (including new edges) between miRNAs and their target genes. PUMA starts with prior knowledge from miRNA target predictions, and then fine-tunes these predictions by comparing with information regarding the co-regulation of the miRNAs’ target genes. It leverages the message passing framework described in our group’s previously developed algorithm, PANDA (Glass *et al.*, 2013), which models regulatory interactions between transcription factors and their target genes by integrating multiple independent sources of data using message passing. This method starts with an initial estimate of the paths of information exchange between regulatory proteins (i.e. transcription factors) and their target genes. It then iteratively refines this prior network by incorporating gene expression and protein–protein interaction data, which provide information on the regulation of genes and on cooperative regulation by transcription factors, respectively. This iterative process allows PANDA to both remove potential false positives from the initial target predictions, and to learn new edges. Since developing PANDA, we have used it to identify differences in transcriptional regulation between multiple human tissues (Sonawane *et al.*, 2017), tissues and their cells-of-origin (Lopes-Ramos *et al.*, 2017), ovarian cancer subtypes (Glass *et al.*, 2015) and to identify sexual dimorphic gene regulation in colon cancer (Lopes-Ramos *et al.*, 2018), among others (Glass *et al.*, 2014; Lao *et al.*, 2015; Vargas *et al.*, 2016; Young *et al.*, 2019).

While PUMA leverages the message passing framework used in PANDA, we introduced several critical modifications to incorporate the effects of miRNAs as an additional class of regulators into the gene regulatory network model. This modified message passing algorithm allows us to effectively integrate miRNA target predictions (either alone or alongside transcription factor regulatory predictions, see Supplementary Methods) with gene expression levels. To illustrate PUMA, we applied the algorithm to model miRNA regulatory networks for 38 tissues from the Genotype-Tissue Expression (GTEx) project, integrating miRNA target predictions with gene expression data for each of the 38 tissues. We built two different collections of networks, each based on a prior obtained from a popular resource of miRNA target predictions, either TargetScan (Agarwal *et al.*, 2015) or miRanda (John *et al.*, 2004)—two resources that have been widely used due to their user-friendliness, maintenance and options to directly download all target predictions for a given species (Peterson *et al.*, 2014). We extracted tissue-specific gene regulation by miRNAs, as well as miRNA functions from these two collections of networks. We found that PUMA consistently captures tissue-specific gene regulation by miRNAs, even when using different input sources of target predictions. Finally, we provide a new resource of tissue-specific functions of miRNAs identified with PUMA and validate predicted tissue-specific functions in a database of disease-associated single nucleotide polymorphisms (SNPs) in miRNA target sites.

## 2 Materials and methods

### 2.1 The PUMA algorithm

We developed PUMA, a regulatory network reconstruction method to model miRNA–target gene interactions. PUMA models these interactions by integrating a regulatory prior with gene expression data. It uses an iterative message passing approach to model information flow between the different data types, finding ‘agreement’ between data represented by multiple networks. For details on the PUMA algorithm and on how PUMA learns miRNA–mRNA edges, please refer to the Supplementary Methods and Figure S1.

### 2.2 GTEx RNA-Seq data

We downloaded RNA-Seq data from the GTEx project (version 6.0, phs000424.v6.1, released October 5, 2015) from dbGap (approved protocol #9112) and used our previously described method YARN (Paulson *et al.*, 2017) to perform quality control and data normalization. YARN removed samples with sex-misidentification and merged related sub-tissues, resulting in a dataset of 9435 gene expression profiles in 38 tissues from 549 individuals. We used default settings in YARN to perform gene filtering for mRNAs (retaining genes with >1 CPM in at least 18 samples), but included expression values for all pre-miRNAs. We then used YARN to perform tissue-aware normalization using qsmooth (Hicks *et al.*, 2018). For more information see Supplementary Methods.

### 2.3 Regulatory network reconstruction

We downloaded miRNA target predictions from TargetScan (Agarwal *et al.*, 2015) and miRanda (John *et al.*, 2004). We selected miRNAs and target genes that were present in both datasets, for which expression levels were available in the GTEx RNA-Seq data (see Supplementary Methods). We used the MATLAB version of PUMA to integrate target predictions from TargetScan and miRanda with gene expression data from each of the 38 GTEx tissues. In total, we modeled 76 gene regulatory networks, two for each tissue. The reconstructed networks are available on Zenodo (doi: 10.5281/zenodo.1313768; https://tinyurl.com/puma-gtex).

### 2.4 Comparison of tissue-specific edges

PUMA returns complete, bipartite networks with edge weights similar to *z*-scores. To compare the tissue-specificity of network edges, we calculated a tissue-specific edge score, which was defined as the deviation of an edge weight $\left(w_{ij}^{\left(t\right)}\right)$ between a miRNA (*i*) and a target gene (*j*) in a particular tissue (*t*) from the median of its weight across all tissues, using the interquartile range (IQR) (as in Sonawane *et al.*, 2017):

(1) $$s_{ij}^{\left(t\right)}=(w_{ij}^{\left(t\right)}-\text{med}(w_{ij}^{\left(\text{all}\right)}))/\text{IQR}(w_{ij}^{\left(\text{all}\right)}).$$

We defined an edge with a specificity score $s_{ij}^{\left(t\right)}>2$ as specific to tissue *t* and the multiplicity of an edge as the number of tissues it is specific to:

(2) $$m_{ij}=\sum_{t}\;[s_{ij}^{\left(t\right)}>2].$$

Similarly, to determine tissue-specific expression levels of miRNAs, we compared the median expression level $\left(e_{p}^{\left(t\right)}\right)$ of a miRNA (*p*) in a particular tissue (*t*) to the median and IQR of its expression levels across all tissues:

(3) $$s_{p}^{\left(t\right)}=(\text{med}(e_{p}^{\left(t\right)})-\text{med}(e_{p}^{\left(\text{all}\right)}))/\text{IQR}(e_{p}^{\left(\text{all}\right)}).$$

We assessed the overlap of the initial TargetScan and miRanda gene regulatory priors with the Jaccard index and with Pearson correlation. We compared tissue-specific edge scores from networks reconstructed on the two different priors using Pearson correlation.

### 2.5 Gene set enrichment analysis on miRNA targeting profiles

For each miRNA in a given tissue, we selected its tissue-specific targeting profile, defined as the tissue-specificity scores of all genes based on their estimated connection to that miRNA. We ran a pre-ranked gene set enrichment analyses (GSEA) (Subramanian *et al.*, 2005) on these profiles to test whether miRNAs specifically target gene ontology (GO) terms in the different tissues. We ran GSEA for networks reconstructed on the TargetScan prior, as well as for networks reconstructed on the miRanda prior. Thus, in total, we ran 48 868 GSEA analyses. For each miRNA/tissue pair, we calculated tissue-specific GO term enrichment scores (ESs), which we defined as the $-{\text{log}}_{10}\text{FDR}$ (false discovery rate) from GSEA, multiplied by the sign of the GSEA ES—those with ES <0 were multiplied by −1, those with ES >0 were multiplied by 1. We then used Pearson correlation across all 733 tissue-specific GO term ESs for each miRNA/tissue pair to assess the similarity of tissue-specific regulation of biological processes by miRNAs.

### 2.6 Community structure analysis to identify sets of related tissue/miRNA–GO terms

We selected highly significant (FDR < 0.001) and positively enriched (ES > 0.65) associations from these analyses and converted these scores into a binary matrix. We then used fast-greedy community detection (Clauset *et al.*, 2004) on this matrix to cluster the data and to identify communities or network modules that share tissue-specific regulatory patterns. We used the Jaccard index to compare nodes (miRNA/tissues and GO terms) that belonged to communities that included at least five GO terms in either the TargetScan or the miRanda networks. We used word clouds to visualize the tissue-specific functions of miRNAs in these communities (see Supplementary Methods).

## 3 Results

### 3.1 Tissue-specific gene regulation by miRNAs

We started by reconstructing miRNA–target gene genome-wide regulatory networks for a large collection of human tissues. We downloaded RNA-Seq data for 54 different tissues (including three different cell types) using Bioconductor package YARN (Paulson *et al.*, 2017). Within YARN, we performed quality control and normalization of the data, merging tissues with similar expression profiles (see Section 2). This resulted in a gene expression dataset that included 9435 samples across 38 tissues. We limited network reconstruction to only those genes and miRNAs that were expressed and which appeared in the TargetScan and miRanda prior, leaving 16 161 genes and 621 miRNAs; these 621 target miRNAs corresponded to 643 regulators in the prior networks (see Supplementary Methods). We then used PUMA to integrate target gene co-expression information for each tissue with an initial regulatory network, which we based on miRNA target predictions from either TargetScan or miRanda. Consequently, our analysis provides two alternative miRNA-mediated gene regulatory networks for each of the 38 tissues (tissue networks), one based on the TargetScan prior and the other alternative based on the miRanda prior. These networks consist of nodes—miRNAs and target genes—and edges for each miRNA–target gene pair that are weighted to represent the likelihood of an interaction between a miRNA and a target gene.

**Fig. 1.**
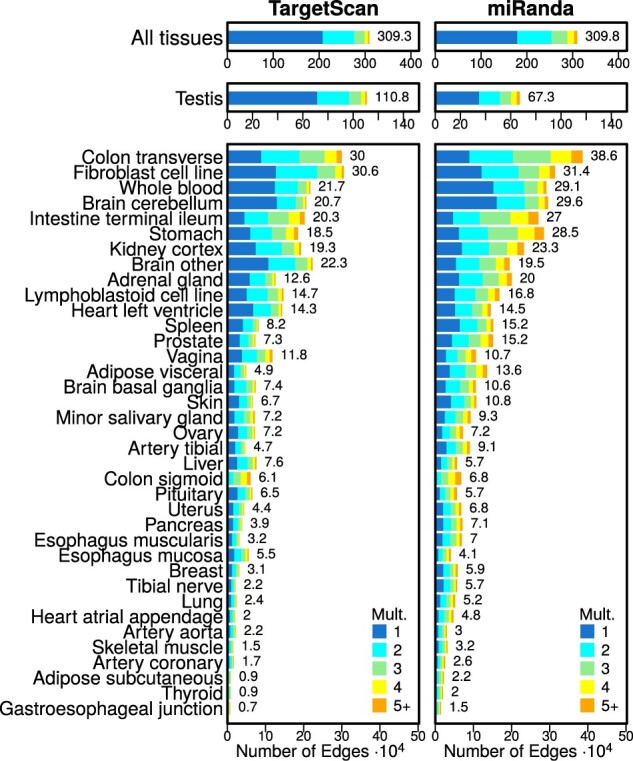
Bar plots illustrating the number of edges modeled on the TargetScan and miRanda priors. The number of elements identified as specific in each tissue is shown to the right of each bar. Tissues are ordered by the average number of tissue-specific edges. Mult.: the edge multiplicity, or the total number of tissues an edge is specific to

We tested for tissue-specific edges in these networks. We defined an edge to be tissue-specific if its weight in a given tissue network was larger than twice the IQR of its weight across all 38 networks (see Section 2). We identified a similar number of tissue-specific miRNA–target gene regulatory edges in the networks modeled on the two different priors—3.093 million and 3.098 million edges for networks modeled on the TargetScan and miRanda prior, respectively (see Fig. 1). In addition, the proportion of tissue-specific edges identified in the different tissues was comparable between the networks modeled on the two different priors (Pearson *R *=* *0.92). The proportion of multiplicities, or the number of tissues in which an edge is identified as specific, was also similar between the two different models.

Even though we identified approximately the same total number of tissue-specific edges across all tissues, on average we identified a larger number of tissue-specific edges per tissue in the networks modeled on the miRanda prior (*t*-statistic = 7.1, *P*-value = 6.5 × 10^−10^). However, we found the opposite to be true for testis—the tissue with the greatest number of tissue-specific edges in both priors. In testis, we identified a substantially larger number (1.65 times) of tissue-specific edges in the networks modeled on the TargetScan prior than in the networks modeled on the miRanda prior.

**Fig. 2.**
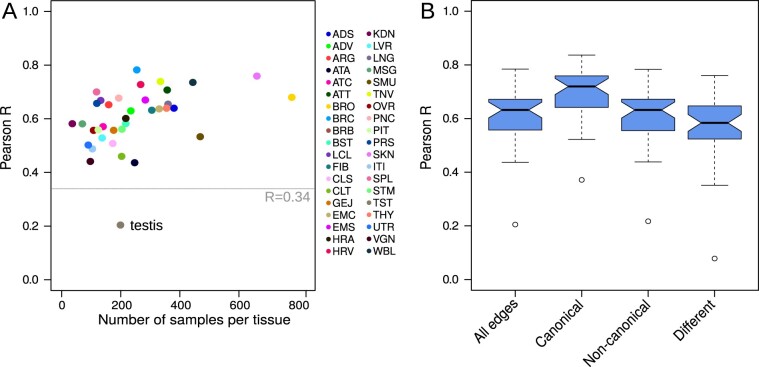
(**A**) Tissue-specificity score similarity—measured using Pearson correlation coefficient (Pearson R)—for each of the 38 miRNA gene regulatory tissue networks modeled on TargetScan and miRanda priors, compared to the number of samples available for each tissue. TargetScan and miRanda priors correlate with *R *=* *0.34. Tissue abbreviations are explained in Supplementary Table S1. (**B**) Boxplots depicting the distribution of edge similarity for all edges, edges that are canonical in both priors, edges that are non-canonical in both priors and edges that are different between the TargetScan and miRanda priors (see Supplementary Fig. S4 for a Venn diagram). Boxplots represent the median and IQR, with whiskers extending out from the box to 1.5× the IQR

We next assessed how similar the edge tissue-specificity scores were between the networks modeled on the different priors. For each of the 38 tissues, we calculated the Pearson correlation coefficient on edge tissue-specificity scores between the tissue network modeled on the two different priors. We found that, in general, PUMA networks modeled using different target predictions result in similar tissue-specificity scores (median Pearson *R *=* *0.63, all *P*-values < 2.2 × 10^−16^, Fig. 2A and Supplementary Fig. S2). This result was stable in subsampling analyses (see Supplementary Methods and Fig. S3). For all tissues, except testis, the resulting PUMA tissue networks modeled on the two different priors were more similar than the two prior networks (which correlated with *R *=* *0.34). This means that, even though there may be differences between various target prediction resources, PUMA helps to fine-tune these predictions into tissue-specific regulatory interactions. We believe that the anomalous gene expression patterns observed in testis, which has been described previously (Sonawane *et al.*, 2017), may, at least in part, be caused by differential targeting by miRNAs.

We then examined the similarity between tissue-specificity scores for miRNA–target gene interactions that were predicted by both TargetScan and miRanda (435 550 ‘canonical’ interactions), interactions that were neither predicted in TargetScan nor in miRanda (8 466 524 ‘non-canonical’ interactions) and edges that were predicted interactions in one of the priors but not in the other (1 489 449 ‘different’, or inconsistent interactions, see also Supplementary Fig. S4). We used Pearson correlation to evaluate the similarity of these different types of edges. We found that, in general, tissue-specificity levels of edges that were canonical in both priors were most reproducible, followed by edges that were non-canonical in both priors. As expected, miRNA–target gene interactions that were canonical in only one of the two priors were less similar compared to edges that were canonical or non-canonical in both priors. However, for those edges the median similarity was still *R *=* *0.58 (Fig. 2B). All Pearson correlations were significant, with *P*-values <2.2 × 10^−16^. This indicates that PUMA can capture consistencies in miRNA–target gene regulation, even when there are inconsistencies between different target prediction resources. It also highlights the strength of modeling miRNA–target gene interactions with PUMA.

### 3.2 Tissue-specific miRNA targeting patterns

**Fig. 3.**
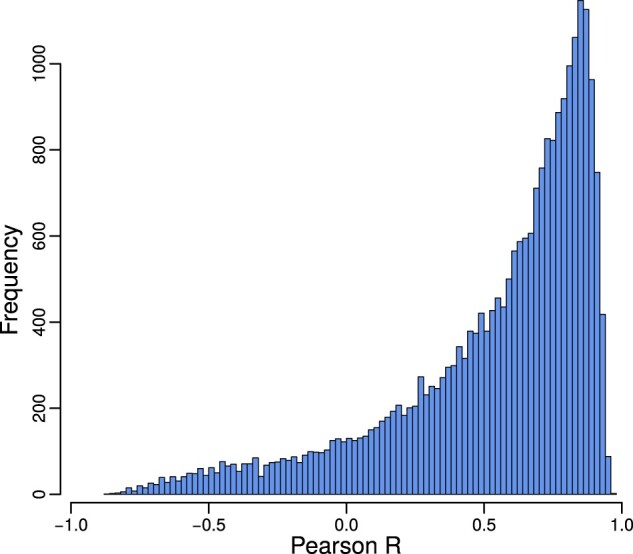
Pearson correlation distribution between the GSEA scores obtained from the 24 434 tissue-specific miRNA targeting profiles computed on TargetScan and the 24 434 profiles computed on the miRanda prior. The bulk of miRNAs have correlating GO term scores in networks modeled using different miRNA priors

To better understand tissue-specific functions of miRNAs, we ran pre-ranked gene set enrichment analysis on the tissue-specific targeting profile of each miRNA in each of the 38 tissues. We did this both for the collection of tissue networks modeled using the TargetScan and for networks modeled using the miRanda priors (see Section 2). We calculated the tissue-specific targeting scores of all 733 available GO terms and investigated whether tissue-specific regulation of biological processes by miRNAs was similar in the two different collections of networks. Most (87.6%) of the miRNA/tissue pairs had a significant (FDR < 0.05) positive Pearson correlation coefficient, with a median Pearson *R* of 0.66 (see Fig. 3 for the correlations between all GSEA scores and Supplementary Fig. S5 for the correlations separated by tissue).

As a negative control, we computed the correlation of miRNA–GO term GSEA scores between different tissues, for the miRanda and TargetScan-generated networks separately. The resulting correlation coefficients were centered around zero, with median Pearson R=−0.002 for the miRanda networks and R=−0.001 for the TargetScan networks, respectively (Supplementary Fig. S6). GSEA scores for miRNA/tissue pairs obtained from the two different collections of networks were significantly more correlated than the negative control (two-group Wilcoxon signed-rank test *P*-value <2.2 × 10^−16^). These results confirm that, even though we used different target predictions as input for PUMA, the actual tissue-specific regulatory functions we obtain from analyzing these networks are highly similar.

**Fig. 4.**
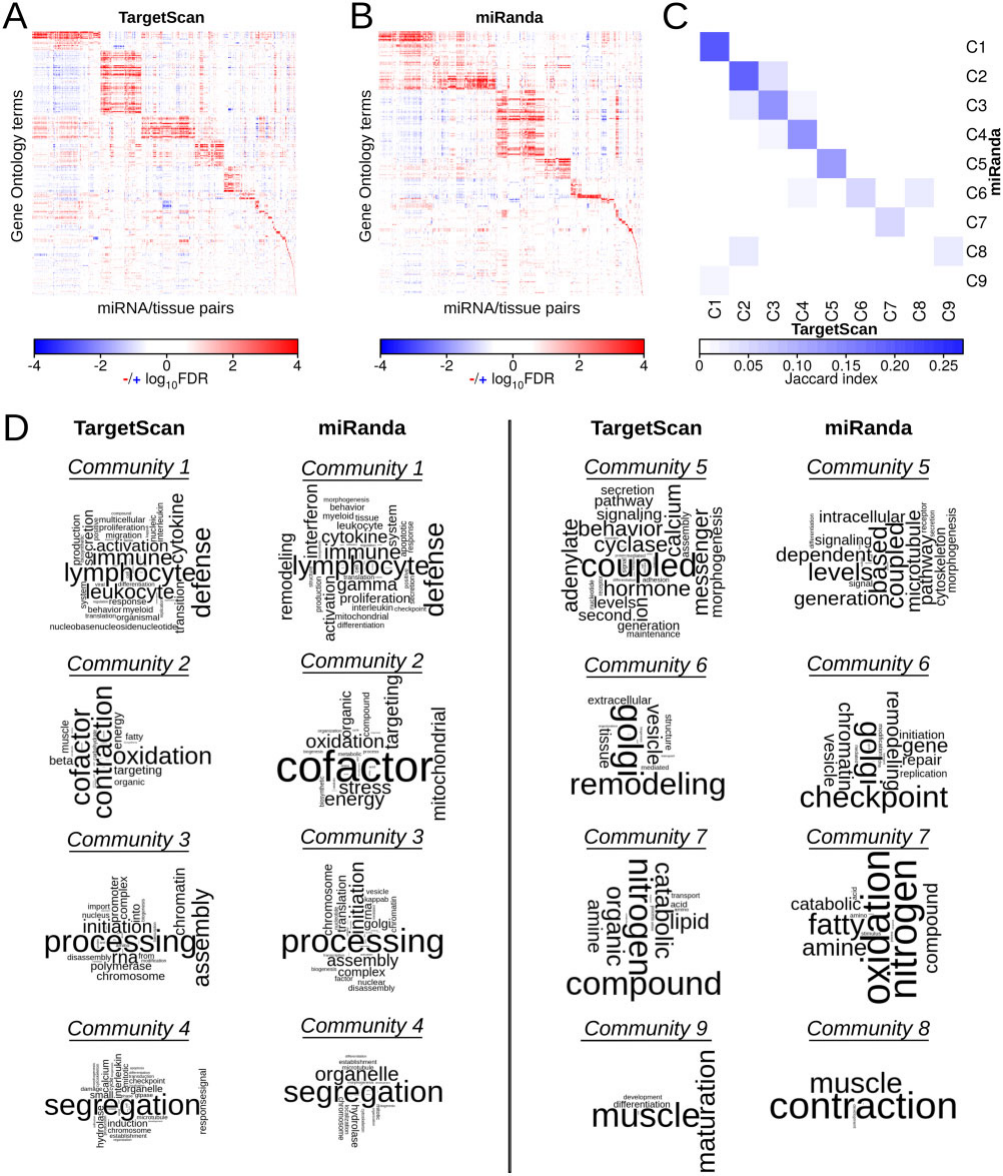
(**A** and **B**) Heatmaps depicting communities of significantly targeted GO terms (FDR < 0.001, ES > 0.65) based on GSEA analyses on all possible miRNA/tissue pairs for the networks modeled on the TargetScan (A) and the miRanda (B) prior. (**C**) Similarity (measured with Jaccard index) of miRNA/tissue–GO term associations in the subset of communities that target at least five GO terms identified in networks modeled using the TargetScan or miRanda prior. Each community is labeled with a number from 1 to 9 and prefix ‘C’. (**D**) Word clouds depicting communities targeting at least five GO terms. Community pairs with the highest Jaccard index are shown. We omitted TargetScan community 8 and miRanda community 9 as they each mapped to communities that corresponded to another community (6 and 1, respectively) with a higher Jaccard index

We tested whether similar miRNA/tissue pairs, as identified in both models, control similar biological functions. To do this, we selected highly significant miRNA/tissue–GO term associations (FDR < 0.001, ES > 0.65), and performed community structure analyses on these sets of associations to identify shared tissue-specific targeting patterns of miRNAs across the tissues (see Section 2). We identified 67 communities (e.g. sets of GO terms grouped together with miRNA/tissue pairs) in the regulatory associations identified in PUMA networks modeled on the TargetScan prior and 64 communities in those identified in PUMA networks modeled on the miRanda prior. The overall modularity of these community structures was 0.76 and 0.77, respectively (see Fig. 4A and B).

In both collections of PUMA tissue networks, nine communities were associated with at least five GO terms. For each of these communities, we calculated the Jaccard index between the two different sets of miRNA/tissue–GO term associations to evaluate the overlap in miRNA/tissue pairs and GO terms associated with the community (Fig. 4C). As can be seen from this figure, the communities have relatively high node overlap, indicating that similar processes are identified as regulated in a tissue-specific manner by similar sets of miRNAs in both analyses. We used word clouds to visualize the biological processes that were associated with these communities, which allowed us to further explore these similarities. Figure 4D shows that similar biological processes are identified as regulated by miRNAs in a tissue-specific manner in these communities. These include processes involved in the immune system, mitochondrial respiration, translation initiation, chromosome segregation, intracellular signaling, protein transport and muscle contraction.

Importantly, we can identify these communities of similarly regulated biological processes in networks modeled using different prior target predictions. This indicates that PUMA’s message passing framework allows us to discover patterns of tissue-specific regulation by miRNAs, even though there may be inconsistencies in the initial target predictions that we used as prior input in PUMA.

### 3.3 A resource of tissue-specific miRNA functions

We compiled a resource of miRNAs that regulate biological processes in a tissue-specific manner. To do this, we took the union of miRNA/tissues significantly regulating GO terms in the TargetScan and the miRanda networks (8992 miRNA/tissue–GO terms in total). We then subsetted this list to only those miRNA/tissues for which the tissue-specific targeting profiles correlated with *R* >0.8 (2085 miRNA/tissue–GO terms). This list of significant tissue-specific functions of miRNAs contained 423 regulator miRNAs, 37 tissues (no consistent tissue-specific regulation was identified for tibial nerve) and 174 GO terms. This resource can be accessed at https://kuijjer.shinyapps.io/puma_gtex/.

**Fig. 5.**
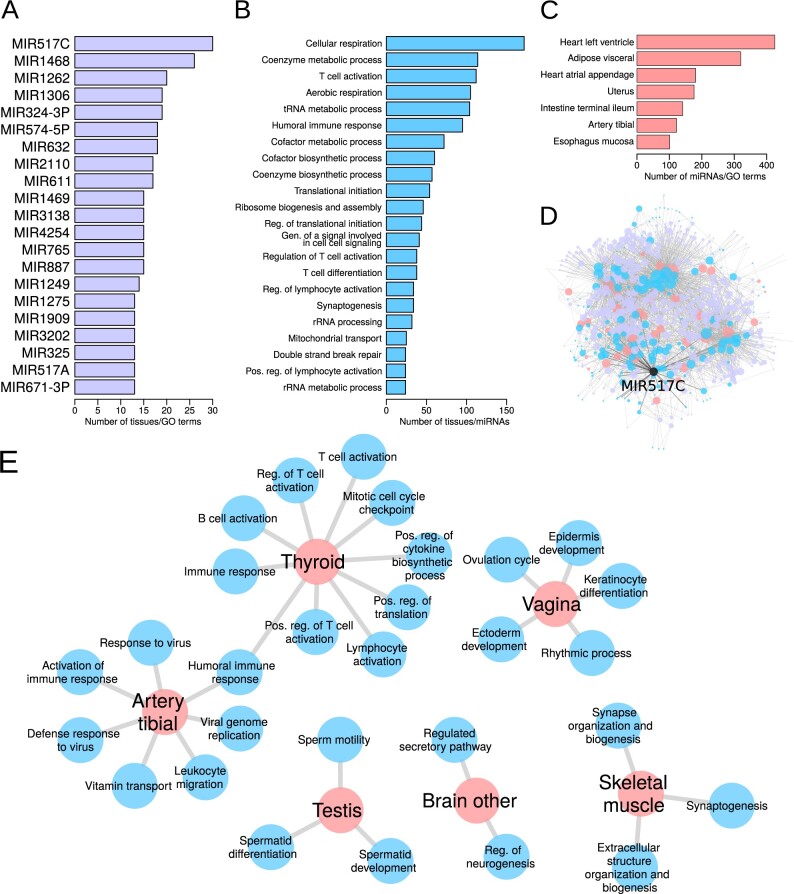
Over-represented miRNAs (**A**), GO terms (**B**) and tissues (**C**) in the database of significant tissue-specific functions of miRNAs. (**D**) Visual representation of the significant tissue-specific regulatory functions of miRNAs present in the Shiny database. Node color illustrates tissues (peach), GO terms (cyan) and miRNAs (magenta). Node size corresponds to the node’s degree. MIR517C—the miRNA with the largest over-representation in tissue-specific connections—is highlighted in black. (**E**) Significant tissue-specific connections made by MIR517C. Gen.: generation; Pos.: positive; Reg.: regulation.

We assessed over-representation of miRNAs, GO terms and tissues in this resource of significant interactions (Fig. 5A–C). Twenty-one miRNAs were over-represented (>median+2·IQR) in regulating multiple tissue-specific processes (Fig. 5A). MIR517C and MIR1468 were the two miRNAs with the highest number of associations of tissue-specific regulation of biological processes (Fig. 5A). MIR517C was associated with immune system processes in tibial artery and thyroid, with ‘regulation of neurogenesis’ and ‘regulated secretory pathway’ in ‘brain other’ (which is composed of multiple brain regions), with synapse-associated processes and ‘extracellular structure organization and biogenesis’ in skeletal muscle, with sperm-associated pathways in testis and with ‘ovulation cycle’ and ectoderm-associated pathways in vagina (Fig. 5D–E). This miRNA has been detected in maternal plasma. It was also recently described to be overexpressed in parathyroid carcinoma (Hu *et al.*, 2018) and to inhibit autophagy and epithelial-to-mesenchymal transition in glioblastoma, a malignant brain cancer (Lu *et al.*, 2015), indicating that deregulation of the expression of this miRNA in tissues in which it regulates tissue-specific processes may lead to cancer.

MIR1468 was associated with ‘sperm motility’ in testis, with many immune system processes in thyroid and with ‘double-strand break repair’ and chromatin-associated processes in whole blood. This miRNA has been implicated in different cancer types (Daniel *et al.*, 2017; Niu *et al.*, 2012) and the latter pathway may indicate a potential mechanism for this. In fact, MIR1468 was recently shown to promote tumor progression by activating PPAR-*γ*-mediated Akt signaling in hepatocellular carcinoma (Liu *et al.*, 2018).

Twenty-two processes were more often targeted in a tissue-specific manner by miRNAs in more tissues than expected by chance. Most of these processes play a role in respiration and metabolism, immune response and protein translation (Fig. 5B), indicating that miRNAs play an important role in regulating these pathways in a tissue-specific manner in multiple tissues.

Seven tissues received significantly more tissue-specific gene regulation by miRNAs compared to all tissues (Fig. 5C). Tissues receiving most tissue-specific gene regulation by miRNAs include heart left ventricle, adipose visceral and heart atrial appendage. We do not know why these tissues have a higher amount of tissue-specific gene regulation by miRNAs. It may be that these tissues are more highly differentiated than others because of the specialized functions they carry out, and so the elevated miRNA activity represses extraneous functions. This could be a potential new area for research.

### 3.4 miRNAs regulating tissue-specific processes are not differentially expressed

We wanted to evaluate whether tissue-specific regulation by miRNAs was caused by tissue-specific expression of those miRNAs. We identified 423 (66%) miRNAs that regulate biological processes in a tissue-specific manner. These regulator miRNAs were associated with 309 different miRNA genes (see Supplementary Methods). We compared the expression levels of these 309 miRNAs with those of the remaining 312 miRNAs in the expression data, and found that miRNAs regulating biological processes in a tissue-specific manner have overall higher expression levels across all samples (two-sided Wilcoxon rank sum test statistic = 4.35 × 10^12^, *P*-value = 2.2 × 10^−16^).

However, when comparing the tissue-specificity scores of these miRNAs in the tissue in which they regulate biological processes, we did not identify any associations. The mean tissue-specificity score [difference in median expression in tissue-of-interest compared to overall median expression, divided by the IQR, see Equation (3)] of these miRNAs was 0.022 (range−0.820 to 6.811), indicating that these miRNAs were not specifically expressed in the tissue they regulate. While none of the miRNAs met our threshold of tissue-specific underexpression, six miRNAs had tissue-specificity scores larger than 2, suggesting tissue-specific overexpression of these miRNAs. These included MIR142-3P regulating the ‘insulin receptor signaling pathway’ in spleen, MIR1909 regulating ‘coenzyme biosynthetic process’ in testis, MIR200B regulating ‘aerobic respiration’ and ‘cellular respiration’ in pancreas and ‘regulation of muscle contraction’ in prostate, MIR203 regulating ‘translational initiation’ in esophagus mucosa, MIR208A regulating ‘activation of immune response’, ‘adaptive immune response’, ‘humoral immune response’ and ‘synaptogenesis’ in heart atrial appendage and MIR632 regulating ‘spermatid differentiation’ in testis.

These findings are in line with our previous results in transcriptional regulatory networks, in which we identified no clear association between a transcription factor’s expression level and its tissue-specific regulation of biological processes (Sonawane *et al.*, 2017). They are also consistent with our previous finding that modeling transcriptional gene regulatory networks are able to identify biologically relevant differences in regulatory processes even in situations where there is little or no differential expression (Lopes-Ramos *et al.*, 2018). Importantly, our results indicate that analysis of miRNA–mRNA co-expression networks, while potentially informative in identifying co-regulation of miRNA and mRNA expression levels, may miss miRNAs that are not differentially expressed, but that do regulate their targets in a tissue- or disease-specific manner. As we have shown here, such miRNAs can be identified using PUMA.

### 3.5 miRNA functions correspond to disease-associated SNPs

To further validate our findings, we integrated the tissue-specificity scores with miRdSNP, a database of SNPs in the 3′ UTR of human genes (Bruno *et al.*, 2012). To do this, we downloaded the miRdSNP database, converted and matched miRNA names, and intersected miRNAs and target genes present in miRdSNP with those present in our regulatory networks. We then matched diseases listed in miRdSNP to GTEx tissues (manual curation, see Supplementary Table S2). This left us with 24 GTEx tissues for which miRNA–target gene associations with disease were available (a total of 591 miRdSNP associations).

For each of these miRNA–target gene associations, we obtained the miRNA’s top predicted tissue-specific function from our Shiny app (settings $-{\text{log}}_{10}(\text{FDR})>0.8$, highest ES). Significant predictions were available for 537/591 associations. We wanted to investigate if these predicted miRNA functions corresponded to the functions of the associated target genes from miRdSNP. To do this, we used a similarity metric to compare the set of GO terms the target gene matched to with those GO terms matching to genes associated with the predicted miRNA’s function (see Supplementary Methods). This information was available for 387 of the associations (not all target genes from miRdSNP were available in the MSigDb GO term signature file).

We found that the predicted tissue-specific function of the miRNA overlapped with the functions of its target gene from miRdSNP in 334/387 (86%) cases. For example, the pathway with the highest level of tissue-specific regulation by MIR429 in coronary artery was ‘endothelial cell migration’. The gene associated with the disease edge from miRdSNP was *VEGFA*, which encodes for a receptor important in angiogenesis. MIR140-5p specifically regulates ‘acute inflammatory response’ in sigmoid colon. This miRNA was associated with disease SNPs in *TLR4*, a receptor involved in innate immunity. In ovary, MIR429 specifically targets ‘G1 phase of mitotic cell cycle’ and was associated with SNPs in *CDK2*, a cell division gene. MIR1197 specifically targets ‘icosanoid metabolic process’ in pancreas and had disease-associated SNPs in *ADIPOR2*, a gene involved in glucose and lipid metabolism. All disease-associated edges are listed in Supplementary Table S3.

These results indicate that the tissue-specific functions of miRNAs predicted using PUMA are important for maintaining tissue homeostasis, and that disrupting miRNA–target gene edges in the regulatory network can perturb these processes, thereby influencing disease. This highlights the importance of modeling genome-wide miRNA–target gene regulatory networks in human tissues.

## 4 Discussion

In this article, we describe PUMA, a new method to model gene regulation by miRNAs. PUMA integrates target gene co-expression information with initial target predictions, which can be obtained from (but are not limited to) resources such as TargetScan or miRanda. We applied PUMA to a large-scale RNA-Seq dataset from GTEx to identify tissue-specific regulatory patterns of miRNAs. We modeled two different collections of tissue networks by integrating gene expression data from GTEx with two prior datasets—target predictions from TargetScan and miRanda, two of the most widely used miRNA target prediction resources. We found that tissue-specific gene regulation by miRNAs was reproducible for most tissues, except for testis. Potentially, the aberrant gene expression pattern in testis is, at least in part, caused by differential regulation by miRNAs. While tissue-specificity of gene regulation was reproducible for different types of edges, it was highest for edges that were predicted in both priors, indicating that compendium-like approaches using the intersection of different miRNA target prediction resources as prior data for network modeling could result in more accurate results. Further evaluation would be needed to determine whether combining target prediction resources would help the accuracy of PUMA. While this is beyond the scope of this study, it could be a future extension of the method.

We performed high-throughput GSEA on the tissue-specific targeting profiles of each of the miRNAs to characterize tissue-specific regulation of biological processes. We found that tissue-specific regulation of biological processes by miRNAs was highly similar in the networks modeled on different priors. We highlighted biological processes that were regulated in a tissue-specific manner (by different sets of miRNAs) in multiple tissues (Fig. 4). The processes we identified play a role in the immune system, mitochondrial respiration, translation initiation, chromosome segregation, intracellular signaling, protein transport and muscle contraction. In addition, we identified miRNAs and tissues for which we found an over-representation of tissue-specific regulation. The most enriched tissue-specific pathways contained genes that were associated with tissue-specific disease-risk SNPs in their 3′ UTR. This highlights the strength of using PUMA networks to identify disease-related genes.

Another strength of PUMA is that it does not use correlations between miRNA expression levels and their target genes to model gene regulation. One of the reasons for not implementing correlation between regulators and their targets as an input in PUMA is that we have previously observed that a regulator’s expression level is often not associated with its regulatory potential (Sonawane *et al.*, 2017), possibly due to combinatorial regulation of the target genes by multiple factors. The analysis presented in the current study again strengthens this. We believe that, while a miRNA needs to be expressed in order to regulate a target gene, the regulatory patterns of an miRNA are complex and depend not only on the miRNA’s expression level, but also on the entire collection of miRNAs that are available in a cell (Memczak *et al.*, 2013), as well as on the complete set of target mRNA transcripts that are expressed.

A good strategy to integrate PUMA networks with miRNA expression data is to overlay the network nodes with miRNA and target gene mRNA expression levels *after* the edges have been estimated with PUMA. This way, one would first identify tissue- or disease-specific edges, and then assess whether these are connected to highly or differentially expressed miRNAs. In fact, we recently used a similar approach to identify tumor suppressor genes downregulated by a cluster of non-coding elements, which had been associated with patient outcome in osteosarcoma (Hill *et al.*, 2017).

A limitation of PUMA is that, like other network reconstruction algorithms, it requires a large number of transcriptomic samples to model an accurate gene regulatory network, but does so only by averaging over a population. As a result, regulatory signals specific to subpopulations might be lost and between-group comparisons are reduced to comparisons of average edge weights. One could instead model single-sample networks by using PUMA in conjunction with our mathematical tool LIONESS. LIONESS uses linear interpolation to extract networks for individual samples from an aggregate network model and has been shown to reconstruct reproducible networks on datasets with as few as 20 samples (Kuijjer *et al.*, 2019). Using PUMA with LIONESS to extract single-sample networks would allow us to perform more robust statistical tests of differences between biological states and might allow identification of new miRNA-based disease subtypes and identify miRNAs that have a subtype-specific regulatory effect.

Gene regulation is a complex process involving multiple factors, including both transcription factors and miRNAs. Understanding these regulatory processes, and how they change between phenotypes, helps elucidating the network changes that occur between health and disease. Identifying genes that are differentially regulated, but not necessarily differentially expressed, can help us to understand the likely potential that a given biological state has to respond to changes, including drug treatment or disease progression. Although there have been many attempts to model gene regulation by transcription factors, few methods have tackled miRNA regulation.

PUMA models gene regulation by miRNAs in a principled way by incorporating our understanding of the regulatory processes that control gene transcript levels. In applying PUMA to a wide variety of tissues, we find patterns of miRNA regulation associated with a variety of tissue-specific processes. As such, PUMA provides the first robust computational method for modeling complex patterns of regulation involving miRNAs. Its implementation is freely available in open-source code, allowing the method to be broadly applied to the analysis of other phenotypes and disease states.

## Supplementary Material

btaa571_supplementary_data
