# Supplementary material for: PUMA: PANDA Using MicroRNA Associations
Source: Bioinformatics. 2020 Jun 17;36(18):4765–73. doi: 10.1093/bioinformatics/btaa571 (PMC7750953; doi:10.1093/bioinformatics/btaa571)
Supplement: btaa571_supplementary_data [file btaa571_supplementary_data.zip › supplement_revision.pdf]

SUPPLEMENTAL METHODS

The PUMA algorithm

PUMA starts with a regulatory prior (W) of initial regulator-target gene interactions. These regulators can be either miRNAs or transcription factors (TFs). Initial regulatory interactions can be combined from multiple sources, such as miRNA-target predictions (for putative interactions of mRNAs by miRNAs), a TF motif scan (for estimated mRNA regulation by a TF), or ChIP-Seq data (for *in vivo* estimates of mRNA regulation by a TF). PUMA also uses a co-regulatory prior (C) of target gene co-expression levels measured using Pearson correlation on gene expression data, and has an optional input of initial TF-TF interactions, which can be based on known or predicted protein-protein interactions (PPIs). These PPIs are overlayed on an identity matrix that includes all regulators (*i.e.* all TFs and all miRNAs), resulting in co-operativity prior P.

PUMA performs a double z-score normalization on these three prior networks, and then quantifies the "agreement" between the different data types using a modified Tanimoto similarity score (T) [1], which evaluates the similarity between sets of interactions in two networks:

$$\begin{aligned} T_{xy} &= T(\vec{x}, \vec{y}) \\ &= \frac{\vec{x}\cdot\vec{y}}{\sqrt{||\vec{x}||^2 + ||\vec{y}||^2 - |\vec{x}\cdot\vec{y}|}} \\ &= \frac{\sum_k x_k y_k}{\sqrt{\sum_k x_k^2 + \sum_k y_k^2 - |\sum_k x_k y_k|}}. \end{aligned} \quad (1)$$

This score is used to calculate the "responsibility" (R) of an edge between a regulator i and a gene j at iteration step t. The responsibility estimate represents information flowing from a regulator to a target gene, and returns a confidence score for how strongly the target gene is regulated by this regulator, taking into account other potential regulators of the gene. PUMA uses protein-protein interaction information to estimate the cooperation between pairs of transcriptional regulators (TFs), and self-interactions to measure the responsibility by miRNAs, using the following equation:

$$R_{ij}^{(t)} = T(P_{i.}^{(t-1)}, W_{.j}^{(t-1)}). \quad (2)$$

In a similar manner, the "availability" (A) estimate represents information flow from a target gene to a regulator and is based on the level of agreement between the targets of a regulator and the set of genes with which the target gene is co-regulated:

$$A_{ij}^{(t)} = T(W_{i.}^{(t-1)}, C_{.j}^{(t-1)}). \quad (3)$$

The initial regulatory network (W) is then updated using an update parameter α (with $0 < \alpha < 1$):

$$W_{ij}^{(t)} = (1-\alpha)W_{ij}^{(t-1)} + \alpha/2(A_{ij}^{(t)} + R_{ij}^{(t)}). \quad (4)$$

This is followed by updating the P and C networks. For each regulator (i), PUMA checks whether it matches with an entry in the input list of miRNAs (q). Since the only interactions a miRNA makes are through regulation of their targets (they do not act in complexes with other miRNAs or TF proteins), P will not be updated between any miRNA-TF interactions or between miRNA-miRNA interactions (except for self-interactions):

$$P_{im}^{(t)} = \begin{cases} P_{im}^{(t-1)} & \text{for } i \in q \vee m \in q \\ (1-\alpha)P_{im}^{(t-1)} & \\ +\alpha T(W_{i.}^{(t)}, [W_{m.}^{(t)}]') & \text{otherwise} \end{cases} . \quad (5)$$

$$C_{kj}^{(t)} = (1-\alpha)C_{kj}^{(t-1)} + \alpha T(W_{.k}^{(t)}, [W_{.j}^{(t)}]'). \quad (6)$$

We note that self-interactions in P (including those among miRNAs) and C are then separately updated in order for the algorithm to converge, as in Glass *et al.* [2, 3]. These message passing steps are repeated until the regulatory network converges.

The message passing framework presented here and used by PUMA, is highly similar to the one from the PANDA algorithm [2, 3]. However, it includes several critical adjustments to account for the mechanisms of miRNA regulation. In particular, we have modified both the initial co-operativity network and its update (as we show in Equation (5)) to account for the different types of regulatory behaviors of TFs and miRNAs. For more details on the message passing algorithm we implemented in PUMA, please refer to Glass *et al.* [2, 3].

PUMA is available in C++ and MATLAB code at `https://github.com/kuijjerlab/PUMA`, and as Python code at `https://github.com/kuijjerlab/PyPuma`.

An intuition on how PUMA learns network edges

The message-passing framework used by PUMA allows us to hypothesize regulation of mRNAs by miRNAs even if a mRNA is not originally a predicted target of a miRNA. In particular, to evaluate a potential novel miRNA-mRNA relationship, PUMA compares the targets of a given miRNA (initialized using target prediction information from a database such as TargetScan of miRanda) with the correlation of those targets with a given mRNA (initialized using mRNA co-expression). If there is a high level of agreement between the targets of a miRNA and their correlation with the selected mRNA, PUMA will add "weight" to the miRNA-mRNA relationship (Supplemental Figure S1). Conversely, a lack of agreement will correspond to a decrease in "weight." This process is then iterated, allowing all the network relationships to adjust until there is optimal agreement between miRNA target predictions and mRNA correlation. This iterative process allows us to both remove potential false positives from the initial miRNA target predictions, and to learn new edges.

GTEx RNA-Seq data

We downloaded the Genotype-Tissue Expression (GTEx) version 6.0 RNA-Seq data (phs000424.v6.p1, 2015-10-05 released) from dbGaP (approved protocol #9112). GTEx release version 6.0 sampled 551 donors with phenotypic information and included 9,590 RNA-Seq assays [4]. We used our previously described method YARN [5] to perform quality control, which removed samples with sex-misidentification and merged related sub-tissues, resulting in a dataset of 9,435 gene expression profiles in 38 tissues from 549 individuals.

We used default settings in YARN to perform gene filtering and tissue-aware normalization using qsmooth [6]. However, only 85 pre-miRNA transcripts were retained after the filtering step in YARN. Because we were particularly interested in using miRNA expression levels to assess the properties of tissue-specific regulator miRNAs that we identified using our networks, we repeated the YARN pipeline without filtering out miRNAs. This resulted in normalized expression levels for 31,384 transcripts, which included 1,136 miRNAs.

To ensure that this procedure did not significantly alter the expression levels obtained with the standard YARN pipeline, we compared expression levels of the 30,248 mRNA and the 85 pre-miRNA transcripts that were not filtered by the standard YARN pipeline with their values in the dataset in which we included all pre-miRNAs. mRNA transcripts correlated with median Pearson $R = 0.9982$, range $[0.9677, 1]$ and the 85 pre-miRNAs correlated with median Pearson $R = 0.9999$, range $[0.9897, 1]$, indicating that adding counts of pre-miRNAs to the count data before the normalization step did not significantly alter the normalized expression levels of other genes.

Pre-processing miRNA target prediction data

We downloaded miRNA target predictions from TargetScan v7.1 (all predictions, file "Summary_Counts.all_predictions.txt.zip," `http://www.targetscan.org/cgi-bin/targetscan/data_download.vert71.cgi`, accessed: July 8, 2016) and miRanda (predictions with "Good mirSVR score, Conserved miRNA," file "human_predictions_S_C_aug2010.txt," `http://34.236.212.39/microrna/getDownloads.do`, accessed: December 5, 2017). We filtered TargetScan interactions by selecting Homo sapiens interactions and by removing interactions with context+ scores larger than -0.1, resulting in 1,524 miRNAs and 18,234 target genes. The miRanda prior contained 1,100 miRNAs and 19,796 target genes.

We selected miRNAs that were present as regulators in both the TargetScan and miRanda priors, and for which expression levels were available. To do this, we first needed to match miRNA identifiers between the different types. We converted miRNA regulator identifiers from TargetScan and miRanda to gene names by changing the character vector to uppercase, removing the "hsa-"prefix, removing the dash character after "LET" and "MIR," and pasting "MIR" in front of miRNAs that start with "LET." We removed all extensions to obtain a list of "base" miRNAs, of which 578 were present as regulators in both prior target prediction resources, and were also available in the expression data.

The numbered suffix in the miRNA identifiers indicate diverse loci that produce identical mature miRNAs. We therefore collapsed these miRNAs in the prior by taking the union of all edges. For duplicates, we selected the most significant edges (lowest context+ score for TargetScan, all edges for miRanda). 643 "regulator" miRNAs corresponded to the set of 578 "base" miRNAs.

An asterisk (*) extension indicates an alternative transcript with lower expression levels. However, information on expression levels of mature miRNAs could have been derived from experiments in specific cell lines or under specific experimental settings, and their expression levels may vary in different tissues. miRNAs with asterisk extensions were only present in miRanda, not in TargetScan. To be able to match these miRNAs between the two different priors, we merged those in the miRanda prior by taking the union of edges.

The -3p/-5p extension in miRNA identifiers indicates whether the mature miRNA product comes from the 3' or 5' end of the hairpin structure formed by the pre-miRNA [7]. This indicates a different mature product with a different seed sequence (that may target different genes). These miRNAs are supposed to have similar expression levels [8], although recent reports identified imbalance in -3p/-5p expression ratios [9]. When evaluating the expression levels of such miRNAs, we used the expression level associated with the miRNA's gene name, so that the same expression level values were assigned to miRNAs from the same genomic location.

This preprocessing resulted in a set of 621 "target" miRNAs for which we had expression data available, which corresponded to the set of 578 "base" miRNAs. Finally, we took the intersection of the lists of target genes in the TargetScan and miRanda regulatory priors with genes for which we had expression data available, resulting in 16,161 target genes.

Subsampling analysis

We performed a subsampling analysis to validate our results on tissue-specific miRNA-gene regulation. For each tissue, we removed a random 20% of the samples before running PUMA. We did this five times without replacement, so that in each fold of the subsampling analysis, a different set of samples was left out of each tissue. We performed this analysis with both the TargetScan and the miRanda priors, resulting in five collections of 38 tissue-networks for each prior. For

each of these subsampled datasets, we calculated tissue-specificity scores (as described in the Methods section of the main text), and calculated Pearson correlation coefficients to assess the reproducibility of the tissue-specificity scores. We found that the results from the subsampling analyses highly matched those from the results modeled on all data (Supplemental Figure S3).

Community structure analysis to identify sets of related tissue/miRNA GO terms

We selected highly significant (FDR < 0.001) and positively enriched (Enrichment Score > 0.65) associations from these analyses and converted these scores into a binary matrix. We then used fast-greedy community detection [10] on this matrix to cluster the data and to identify communities or network modules that share tissue-specific regulatory patterns.

We then used the Jaccard index to compare nodes (miRNA/tissues and GO terms) that belonged to communities that included at least 5 GO terms in either the TargetScan or the miRanda networks. We used word clouds to visualize the tissue-specific functions of miRNAs in these communities. To do this, we split the strings for each of the significant GO term into separate words and removed words that occurred less than 3 times to obtain a background list of word frequencies associated with all significant GO terms. We then counted the number of times a word was present in the community of interest, and divided this by the total number of words associated with significant GO terms in that community (the "observed" rate), as well as the number of times the word occurred in the background list, divided by the total number of words in that background list (the "expected" rate). We then calculated the observed/expected ratio, multiplied this by 10, and rounded this number to an integer to obtain a word occurrence score. Finally, we added the word, repeating it by its word occurrence score, to a list. We used this list of normalized word occurrences as input in `https://www.wordclouds.com/` to generate a word cloud for that community. We repeated this for each of the communities that included tissue-specific targeting by miRNAs of at least 5 GO terms.

miRdSNP analysis

We downloaded the miRdSNP database from `http://mirdsnp.ccr.buffalo.edu/` (download date Nov 11, 2019). We converted and matched miRNA names as described above and intersected miRNAs and target genes present in miRdSNP with those present in our regulatory networks. We then matched diseases listed in miRdSNP to GTEx tissues (manual curation, see Supplemental Table S2). This left us with 24 GTEx tissues for which miRNA-target gene associations with disease were available (a total of 591 miRdSNP associations).

For each of these miRNA-target gene associations, we obtained the miRNA's top predicted tissue-specific function from our Shiny app [11] (settings $-log_{10}(FDR) > 0.8$, highest ES). Significant predictions were available for 537/591 associations. To investigate if these predicted miRNA functions corresponded to the functions of their target genes, we converted the GO term signature from MSigDb .gmt file to a binary matrix M, with information of whether a gene j belongs to a pathway q using the "convertgmt" function from the SAMBAR package (`https://github.com/kuijjerlab/SAMBAR`, see also [12]). This information was available for 387/537 associations, as not all target genes were present in the GO term signature file from MSigDb. For each gene–gene pair, we calculated the Jaccard similarity, representing the similarity in GO terms that two genes belong to. Next, we identified the genes belonging to the miRNA's tissue specific function and, for these genes, calculated the mean Jaccard similarity to the target gene of that specific miRdSNP association. We then repeated this for each miRdSNP association, obtaining a similarity index for biological function of the miRNA and the target gene from miRdSNP. 334/387 associations had similarity index > 0, indicating that there was overlap between the PUMA-predicted tissue-specific function of the miRNA and of the target gene obtained from miRdSNP. All associations are listed in Supplemental Table S3.

Data availability

The reconstructed networks are available on Zenodo (doi: 10.5281/zenodo.1313768; `https://tinyurl.com/puma-gtex`). An R Shiny app [11] that can be used to assess tissue-specific functions of miRNAs using different thresholds is hosted on `https://kuijjer.shinyapps.io/puma_gtex/`.

[1] Rogers DJ, Tanimoto TT. A computer program for classifying plants. Science. 1960;132(3434):1115–1118.

[2] Glass K, Huttenhower C, Quackenbush J, Yuan GC. Passing messages between biological networks to refine predicted interactions. PloS one. 2013;8(5):e64832.

[3] Glass K, Quackenbush J, Kepner J. High performance computing of gene regulatory networks using a message-passing model. In: 2015 IEEE High Performance Extreme Computing Conference (HPEC). IEEE; 2015. p. 1–6.

[4] GTEx Consortium, et al. The Genotype-Tissue Expression (GTEx) pilot analysis: multitissue gene regulation in humans Science. 2015;348(6235)648–660.

[5] Paulson JN, Chen CY, Lopes-Ramos CM, Kuijjer ML, Platig J, Sonawane AR, et al. Tissue-aware RNA-Seq processing and

normalization for heterogeneous and sparse data. BMC bioinformatics. 2017;18(1):437.
[6] Hicks SC, Okrah K, Paulson JN, Quackenbush J, Irizarry RA, Bravo HC. Smooth quantile normalization. Biostatistics. 2018;19(2):185–198.
[7] Ha M, Kim VN. Regulation of microRNA biogenesis. Nature reviews Molecular cell biology. 2014;15(8):509.
[8] miRBase; 2011. [Online; accessed June 21, 2018]. http://www.mirbase.org/blog/2011/04/whats-in-a-name/.
[9] Siddle KJ, Tailleux L, Deschamps M, Loh YHE, Deluen C, Gicquel B, et al. Bacterial infection drives the expression dynamics of microRNAs and their isomiRs. PLoS genetics. 2015;11(3):e1005064.
[10] Clauset A, Newman ME, Moore C. Finding community structure in very large networks. Physical review E. 2004;70(6):066111.
[11] Chang W, Cheng J, Allaire J, Xie Y, McPherson J. shiny: Web Application Framework for R; 2018. R package version 1.1.0. Available from: https://CRAN.R-project.org/package=shiny.
[12] Kuijjer ML, Paulson JN, Salzman P, Ding W, Quackenbush J. Cancer subtype identification using somatic mutation data British journal of cancer. 2018;118(11):1492–1501

SUPPLEMENTAL FIGURES AND TABLES

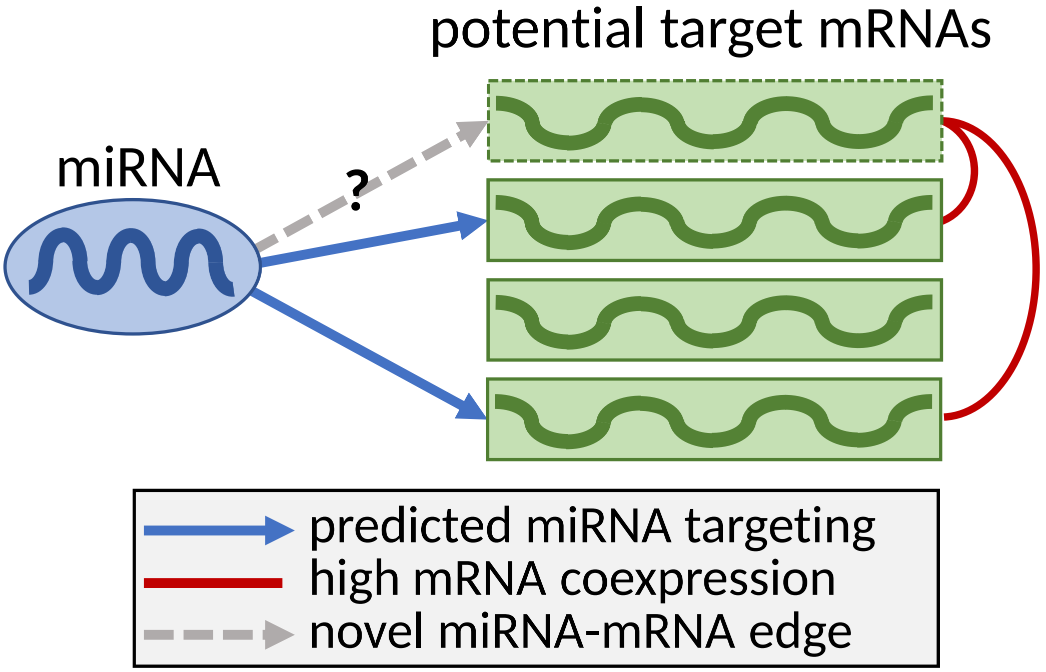

Supplemental Figure S1. Representation of how PUMA can learn novel miRNA-mRNA edges by leveraging information on predicted miRNA targeting and mRNA co-expression. In this example, three genes are co-expressed (indicated with red lines). Two of these genes are predicted to be regulated by a specific miRNA (blue arrows). As genes that are co-expressed are likely to be regulated by the same set of miRNAs, PUMA will increase the edge weight between the miRNA and the top gene, which was initially not a predicted target of that miRNA, and thus learn this new miRNA-mRNA edge.

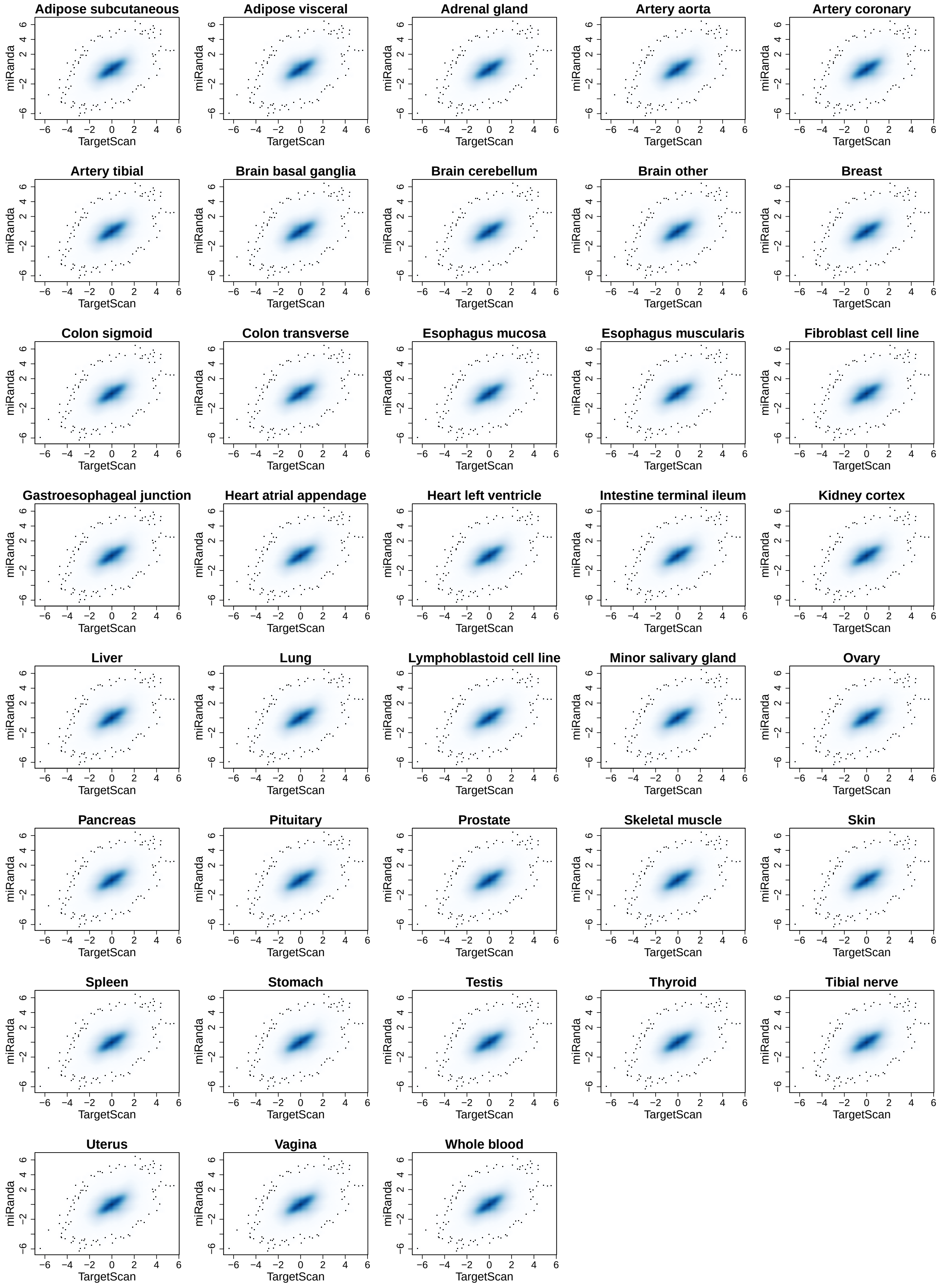

Supplemental Figure S2. Smooth scatterplot depicting, for each tissue, the correlation of all tissue-specificity scores of networks modeled on the TargetScan and the miRanda prior.

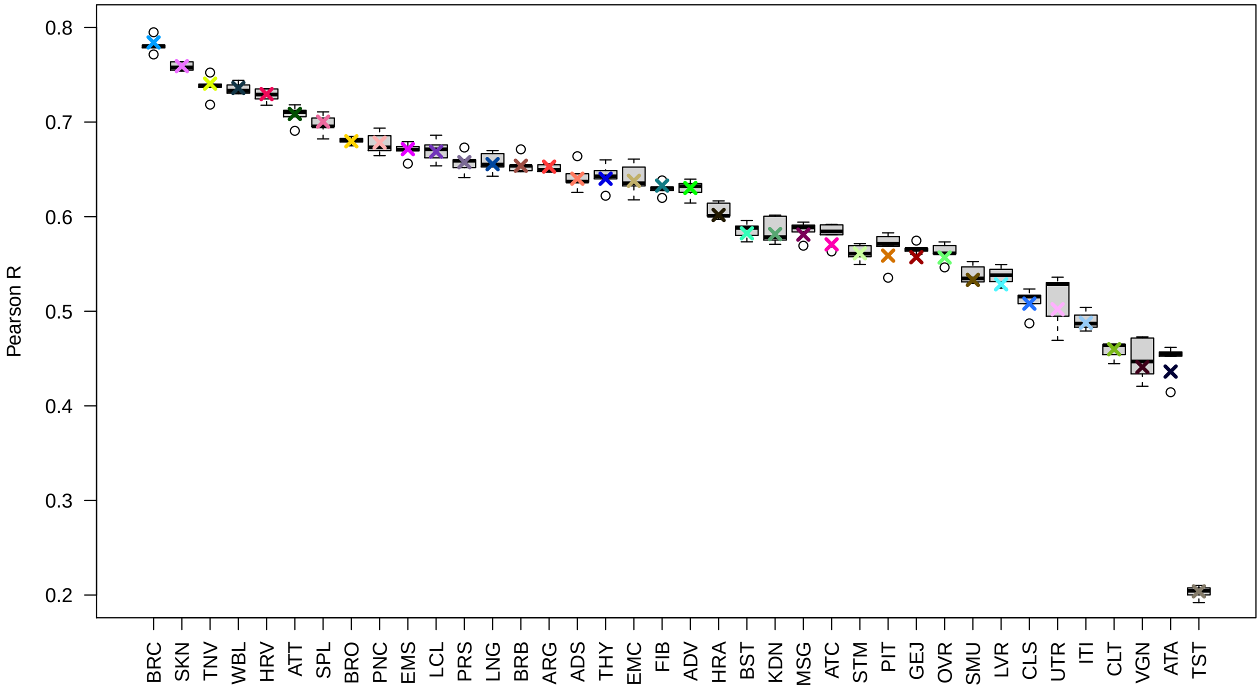

Supplemental Figure S3. Results from the subsampling analysis. For each tissue (see Supplemental Table S1 for descriptions of tissue abbreviations) a boxplot is shown of the distribution of the tissue-specificity score similarities (measured with Pearson correlation) for the networks modeled on the TargetScan and miRanda priors, across the five resampled datasets. Boxplots represent the median and IQR, with whiskers extending out from the box to 1.5× the IQR. The colored crosses show the Pearson R for the analysis on the complete dataset (they are the same values as shown in Figure 2A). The color coding of the colored crosses matches that of the tissue color coding in Figure 2A.

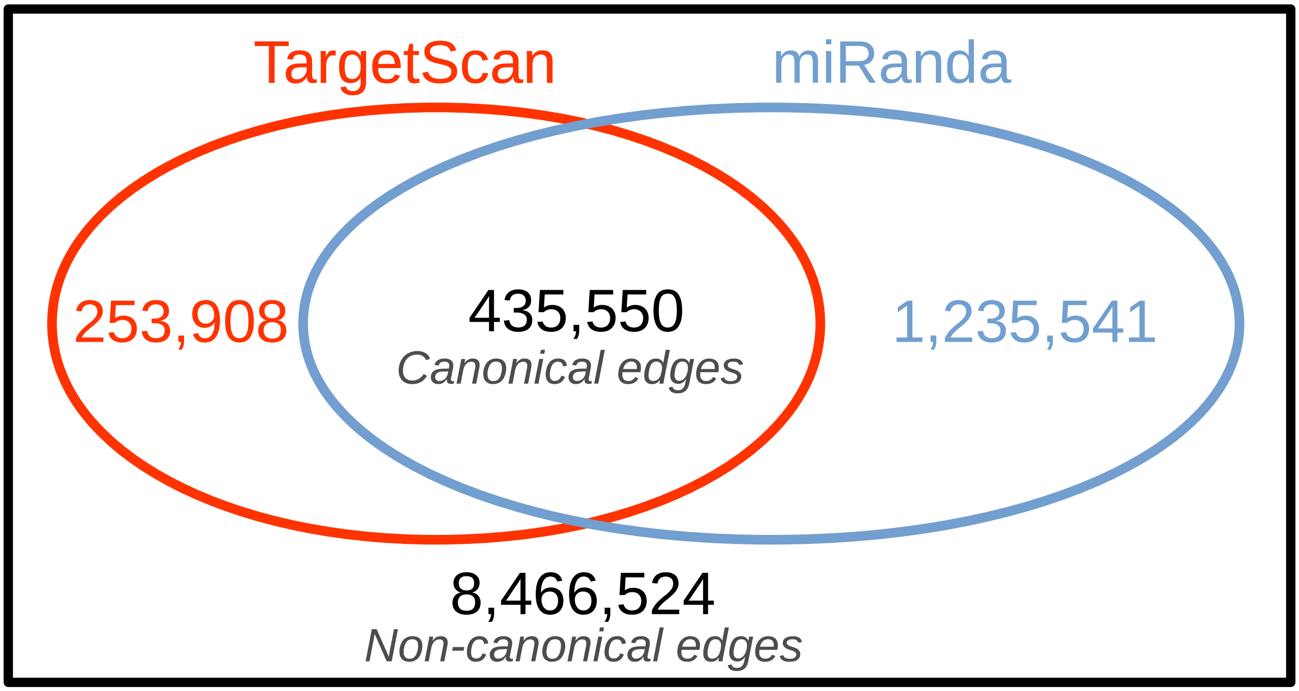

Supplemental Figure S4. Venn diagram depicting the number of overlapping canonical ("prior") and non-canonical edges in the TargetScan and miRanda priors.

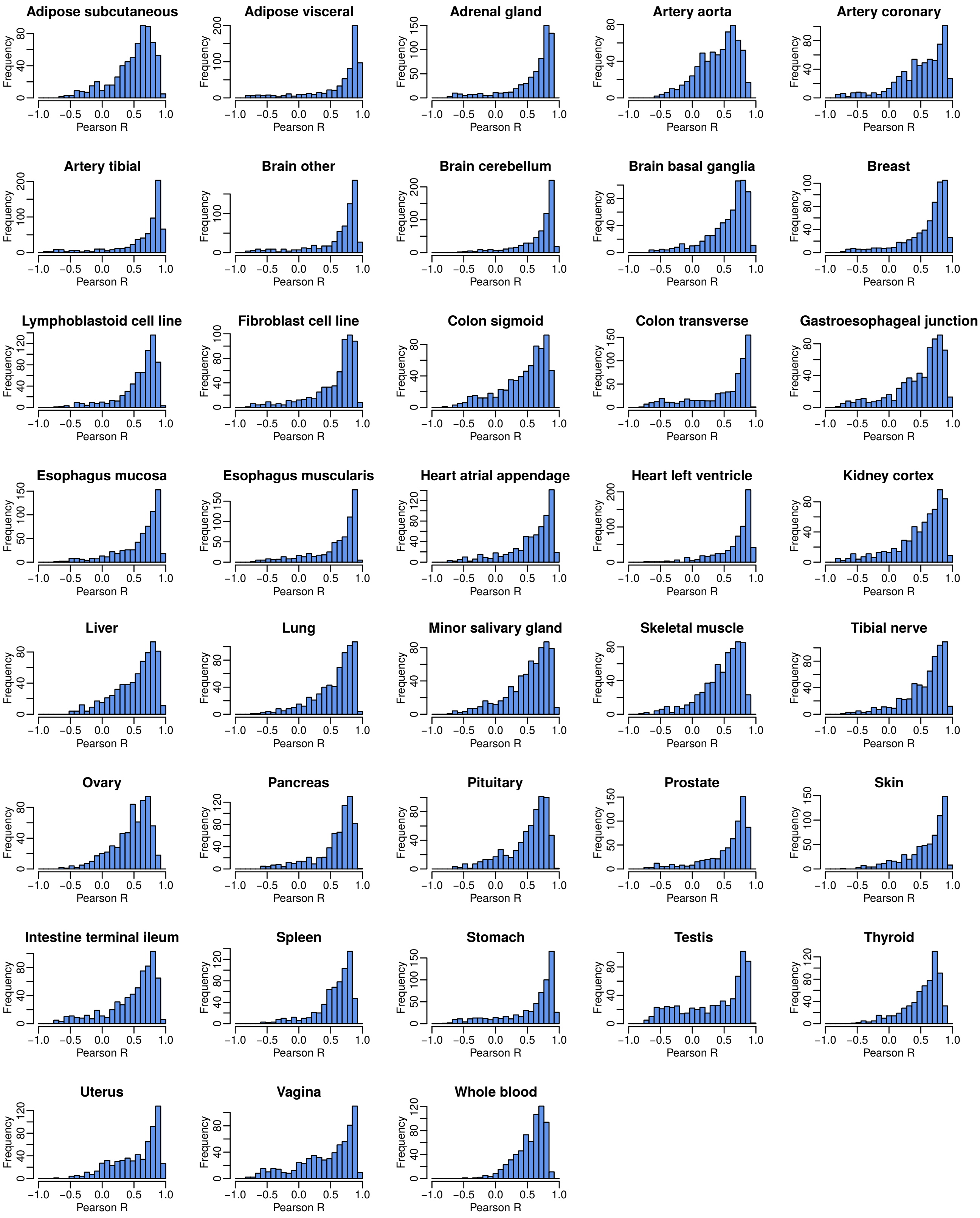

Supplemental Figure S5. Histogram of Pearson correlation coefficients obtained from comparing the GSEA scores of the tissue-specific miRNA targeting profiles computed on the TargetScan and the miRanda prior, visualized for each tissue individually.

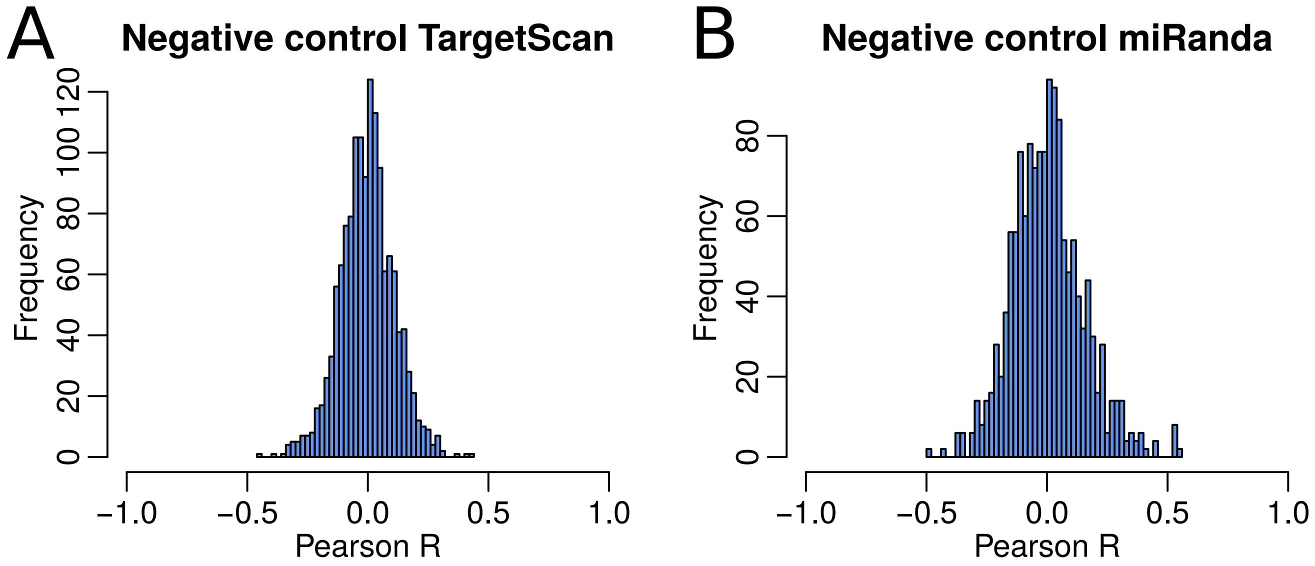

Supplemental Figure S6. Negative control for the similarity analysis of miRNA/tissue GSEA scores predicted on networks obtained from the two different priors (shown in Figure 3). Here, we compared tissue-specific GSEA scores for one miRNA in one specific tissue with those from the same miRNA in all other tissues, using networks modeled on the same prior—either from TargetScan (A) or miRanda (B).

Abbreviation	Tissue
ADS	Adipose subcutaneous
ADV	Adipose visceral
ARG	Adrenal gland
ATA	Artery aorta
ATC	Artery coronary
ATT	Artery tibial
BRO	Brain other
BRC	Brain cerebellum
BRB	Brain basal ganglia
BST	Breast
LCL	Lymphoblastoid cell line
FIB	Fibroblast cell line
CLS	Colon sigmoid
CLT	Colon transverse
GEJ	Gastroesophageal junction
EMC	Esophagus mucosa
EMS	Esophagus muscularis
HRA	Heart atrial appendage
HRV	Heart left ventricle
KDN	Kidney cortex
LVR	Liver
LNG	Lung
MSG	Minor salivary gland
SMU	Skeletal muscle
TNV	Tibial nerve
OVR	Ovary
PNC	Pancreas
PIT	Pituitary
PRS	Prostate
SKN	Skin
ITI	Intestine terminal ileum
SPL	Spleen
STM	Stomach
TST	Testis
THY	Thyroid
UTR	Uterus
VGN	Vagina
WBL	Whole blood

Supplemental Table S1. Tissue abbreviations from Figure 2A explained.

Supplemental Table S2. Diseases from miRdSNP matched to GTEx tissues (tab separated file).

Supplemental Table S3. Results from the miRdSNP validation analysis (tab separated file).
